# 2,2′-Dimeth­oxy-6,6′-dinitro­biphen­yl

**DOI:** 10.1107/S1600536809033790

**Published:** 2009-09-05

**Authors:** Shao-Bin Miao, Dong-Sheng Deng, Xian-Ming Liu, Bao-Ming Ji

**Affiliations:** a College of Chemistry and Chemical Engineering, Luoyang Normal University, Luoyang 471022, People’s Republic of China

## Abstract

In the title compound, C_14_H_12_N_2_O_6_, the half mol­ecule in the asymmetric unit of the cell is completed by a crystallographic twofold rotation axis, and the two benzene rings of the complete mol­ecule make a dihedral angle of 60.5 (3)°. Furthermore, inter­molecular weak C—H⋯O hydrogen bonds link adjacent mol­ecules, forming a two-dimensional sheet. These sheets are stablized by face-to-face weak π–π contacts [centroid–centroid distance = 3.682 (1) Å] between the nearly parallel [dihedral angle = 0.12 (7)°] benzene rings of the neighboring mol­ecules, resulting in a three-dimensional network.

## Related literature

For the synthesis of the title compound, see: Chen *et al.* (2001 ▶). For asymmetric synthesis using chiral ligands with *C*_2_ symmetry, see: Jiang *et al.* (2001 ▶); García *et al.* (2002 ▶). For synthetic methods for chiral compounds, see: Brunel (2005 ▶); Kočovský *et al.* (2003 ▶). For related biphenyl structures, see: Fischer *et al.* (2007 ▶). For related structural data see: Yang *et al.* (2005 ▶).
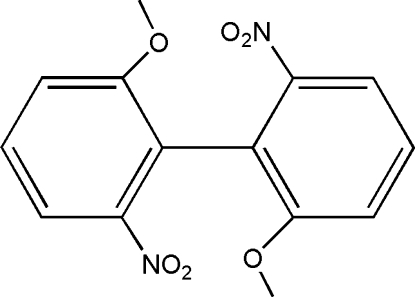


## Experimental

### 

#### Crystal data

C_14_H_12_N_2_O_6_
*M* *_r_* = 304.26
Monoclinic, 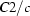

*a* = 18.236 (3) Å
*b* = 7.7826 (12) Å
*c* = 10.9079 (17) Å
β = 115.089 (2)°
*V* = 1402.0 (4) Å^3^
*Z* = 4
Mo *K*α radiation
μ = 0.12 mm^−1^
*T* = 294 K
0.30 × 0.18 × 0.18 mm

#### Data collection

Bruker APEXII CCD area-detector diffractometer
Absorption correction: multi-scan (*SADABS*; Sheldrick, 1996 ▶) *T*_min_ = 0.966, *T*_max_ = 0.979
5102 measured reflections
1298 independent reflections
1009 reflections with *I* > 2σ(*I*)
*R*_int_ = 0.019

#### Refinement

*R*[*F* ^2^ > 2σ(*F* ^2^)] = 0.035
*wR*(*F* ^2^) = 0.097
*S* = 1.03
1298 reflections
101 parameters
H-atom parameters constrained
Δρ_max_ = 0.14 e Å^−3^
Δρ_min_ = −0.13 e Å^−3^

### 

Data collection: *APEX2* (Bruker, 2004 ▶); cell refinement: *SAINT* (Bruker, 2004 ▶); data reduction: *SAINT*; program(s) used to solve structure: *SHELXS97* (Sheldrick, 2008 ▶); program(s) used to refine structure: *SHELXL97* (Sheldrick, 2008 ▶); molecular graphics: *SHELXTL* (Sheldrick, 2008 ▶) and *PLATON* (Spek, 2009 ▶); software used to prepare material for publication: *SHELXL97* and *PLATON*.

## Supplementary Material

Crystal structure: contains datablocks global, I. DOI: 10.1107/S1600536809033790/si2193sup1.cif

Structure factors: contains datablocks I. DOI: 10.1107/S1600536809033790/si2193Isup2.hkl

Additional supplementary materials:  crystallographic information; 3D view; checkCIF report

## Figures and Tables

**Table 1:** Hydrogen-bond geometry (Å, °)

*D*—H⋯*A*	*D*—H	H⋯*A*	*D*⋯*A*	*D*—H⋯*A*
C7—H7*B*⋯O3^i^	0.96	2.48	3.426 (3)	169

Symmetry code: (i) 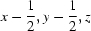.

**Fig. 1.**
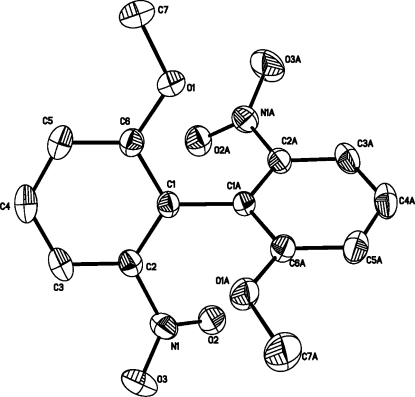
ORTEP drawing (30% probability displacement ellipsoids) of a single molecule of the title compound.

**Fig. 2.**
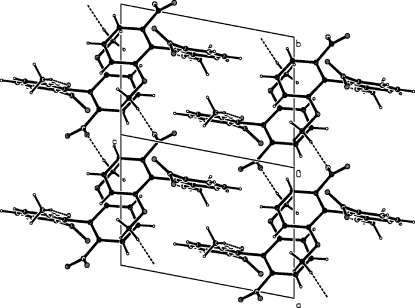
three-dimensional structures of the title compound.

## supplementary crystallographic information

### Comment

A large number of chiral compounds with *C*_2_-symmetry are widely used as chiral auxiliaries and ligands in asymmetric synthesis and have shown high stereocontrol properties in a wide range of asymmetric transformations (Jiang *et al.* 2001; García *et al.* 2002). Design and synthesis of such compounds play a very important role in the development of highly enantioselective asymmetric reactions. Thus, it is not surprising that a lot of methods have been developed to obtain these chiral compounds (Brunel 2005; Kočovský *et al.* 2003). In this paper, we report the synthesis and crystal structure of the title compound with *C*_2_-symmetry.

A view of the molecular structure of the title compound is given in Fig.1. All bond lengths and angles are in the expected range and in good agreement with those reported previously (Yang *et al.* 2005). The dihedral angle between two benzene rings is 60.5 (3)°, which is considerable larger than those found in other biphenyls (Fischer *et al.* 2007), possibly due to the concomitant effects of the steric hindrance of adjacent methoxy and nitro groups.

In the crystal structure, each molecule is connected by four adjacent molecules through intermolecular C—H···O hydrogen bonds (Table 1), between methoxy groups and O atoms of the adjacent nitro groups, leading to the formation of a two-dimensional sheet in the *ac* plane. The sheets are further connected into a three-dimensional network(Fig.2) by the face-to-face weak π–π contacts between nearly parallel benzene rings of the neighboring title molecules. The *Cg*1···*Cg*1^iii^ distance is 3.6823 (11) Å, the perpendicular distance between the rings is 3.410 Å, and the slippage between the rings is 1.389 Å. *Cg*1 is the centroid of the benzene ring C1 - C6, the symmetry code iii = 1 - *x*, -*y*, 1 - *z*.

### Experimental

The title compound was synthesized by a reported method (Chen, *et al.* 2001),namely, a mixture of 2-iodo-3-nitroanisol (14 g, 0.05 mol) and activated copper brone (9.5 g, 0.15 mol), 50 ml of dimethylformamide was stirried at 140°C for 4 h under nitrogen atmosphere. Yellow crystals suitable for X-ray diffraction study were obtained from a solution in acetic ester.

### Refinement

All of the non-hydrogen atoms were refined anisotropically. The hydrogen atoms were assigned with common isotropic displacement factors *U*_iso_(H) = 1.2 times *U*_eq_(C,*N*) and 1.5 times *U*_eq_(O), respectively, and included in the final refinement by using geometrical restraints, with C–H distances of 0.93 Å.

### Crystal data

**Table:**

C_14_H_12_N_2_O_6_	*F*(000) = 632
*M**_r_* = 304.26	*D*_x_ = 1.441 Mg m^−^^3^
Monoclinic, *C*2/*c*	Mo *K*α radiation, λ = 0.71073 Å
Hall symbol: -C 2yc	Cell parameters from 1790 reflections
*a* = 18.236 (3) Å	θ = 2.5–25.7°
*b* = 7.7826 (12) Å	µ = 0.12 mm^−^^1^
*c* = 10.9079 (17) Å	*T* = 294 K
β = 115.089 (2)°	Block, yellow
*V* = 1402.0 (4) Å^3^	0.30 × 0.18 × 0.18 mm
*Z* = 4	

### Data collection

**Table:**

Bruker APEXII CCD area-detector diffractometer	1298 independent reflections
Radiation source: fine-focus sealed tube	1009 reflections with *I* > 2σ(*I*)
graphite	*R*_int_ = 0.019
φ and ω scans	θ_max_ = 25.5°, θ_min_ = 2.5°
Absorption correction: multi-scan (*SADABS*; Sheldrick, 1996)	*h* = −22→22
*T*_min_ = 0.966, *T*_max_ = 0.979	*k* = −9→9
5102 measured reflections	*l* = −13→13

### Refinement

**Table:**

Refinement on *F*^2^	Primary atom site location: structure-invariant direct methods
Least-squares matrix: full	Secondary atom site location: difference Fourier map
*R*[*F*^2^ > 2σ(*F*^2^)] = 0.035	Hydrogen site location: inferred from neighbouring sites
*wR*(*F*^2^) = 0.097	H-atom parameters constrained
*S* = 1.03	*w* = 1/[σ^2^(*F*_o_^2^) + (0.0401*P*)^2^ + 0.8036*P*] where *P* = (*F*_o_^2^ + 2*F*_c_^2^)/3
1298 reflections	(Δ/σ)_max_ < 0.001
101 parameters	Δρ_max_ = 0.14 e Å^−^^3^
0 restraints	Δρ_min_ = −0.13 e Å^−^^3^

### Special details

**Table:**

Geometry. All e.s.d.'s (except the e.s.d. in the dihedral angle between two l.s. planes)are estimated using the full covariance matrix. The cell e.s.d.'s are takeninto account individually in the estimation of e.s.d.'s in distances, anglesand torsion angles; correlations between e.s.d.'s in cell parameters are onlyused when they are defined by crystal symmetry. An approximate (isotropic)treatment of cell e.s.d.'s is used for estimating e.s.d.'s involving l.s. planes.
Refinement. Refinement of *F*^2^ against ALL reflections. The weighted *R*-factor *wR* and goodness of fit *S* are based on *F*^2^, conventional *R*-factors *R* are based on *F*, with *F* set to zero for negative *F*^2^. The threshold expression of *F*^2^ > σ(*F*^2^) is used only for calculating *R*-factors(gt) *etc*. and is not relevant to the choice of reflections for refinement. *R*-factors based on *F*^2^ are statistically about twice as large as those based on *F*, and *R*- factors based on ALL data will be even larger.

### Fractional atomic coordinates and isotropic or equivalent isotropic displacement parameters (Å^2^)

**Table:**

	*x*	*y*	*z*	*U*_iso_*/*U*_eq_	
C1	0.49134 (9)	0.22562 (19)	0.67636 (14)	0.0387 (4)	
C2	0.54245 (9)	0.2962 (2)	0.62577 (15)	0.0434 (4)	
C3	0.52777 (12)	0.2896 (2)	0.49065 (17)	0.0546 (5)	
H3	0.5639	0.3378	0.4607	0.066*	
C4	0.45847 (12)	0.2102 (2)	0.40223 (17)	0.0588 (5)	
H4	0.4471	0.2058	0.3107	0.071*	
C5	0.40569 (11)	0.1370 (2)	0.44656 (16)	0.0540 (5)	
H5	0.3589	0.0835	0.3852	0.065*	
C6	0.42203 (9)	0.1426 (2)	0.58296 (15)	0.0444 (4)	
C7	0.30000 (13)	−0.0071 (4)	0.5464 (2)	0.0944 (8)	
H7A	0.2673	0.0767	0.4817	0.142*	
H7B	0.2716	−0.0485	0.5971	0.142*	
H7C	0.3110	−0.1012	0.4998	0.142*	
N1	0.61559 (9)	0.3880 (2)	0.71661 (15)	0.0557 (4)	
O1	0.37432 (7)	0.06986 (18)	0.63620 (11)	0.0594 (4)	
O2	0.61327 (8)	0.47507 (17)	0.80791 (13)	0.0615 (4)	
O3	0.67594 (9)	0.3739 (3)	0.69543 (17)	0.0967 (6)	

### Atomic displacement parameters (Å^2^)

**Table:**

	*U*^11^	*U*^22^	*U*^33^	*U*^12^	*U*^13^	*U*^23^
C1	0.0399 (8)	0.0425 (8)	0.0345 (8)	0.0057 (6)	0.0166 (7)	0.0011 (6)
C2	0.0464 (9)	0.0454 (9)	0.0426 (8)	0.0044 (7)	0.0229 (7)	0.0023 (7)
C3	0.0722 (12)	0.0549 (10)	0.0508 (10)	0.0071 (9)	0.0399 (9)	0.0053 (8)
C4	0.0853 (14)	0.0566 (11)	0.0369 (8)	0.0117 (10)	0.0283 (10)	0.0036 (8)
C5	0.0610 (11)	0.0545 (10)	0.0372 (8)	0.0046 (9)	0.0119 (8)	−0.0031 (8)
C6	0.0452 (9)	0.0473 (9)	0.0388 (8)	0.0033 (7)	0.0159 (7)	0.0004 (7)
C7	0.0600 (13)	0.142 (2)	0.0750 (14)	−0.0445 (14)	0.0224 (12)	−0.0253 (15)
N1	0.0500 (8)	0.0679 (10)	0.0571 (9)	−0.0031 (7)	0.0302 (7)	0.0073 (8)
O1	0.0473 (7)	0.0794 (9)	0.0474 (7)	−0.0193 (6)	0.0162 (6)	−0.0058 (6)
O2	0.0612 (8)	0.0665 (8)	0.0570 (7)	−0.0133 (6)	0.0253 (6)	−0.0081 (7)
O3	0.0604 (9)	0.1503 (16)	0.0999 (12)	−0.0197 (10)	0.0538 (9)	−0.0120 (11)

### Geometric parameters (Å, °)

**Table:**

C1—C2	1.383 (2)	C5—C6	1.389 (2)
C1—C6	1.400 (2)	C5—H5	0.9300
C1—C1^i^	1.500 (3)	C6—O1	1.3576 (19)
C2—C3	1.383 (2)	C7—O1	1.424 (2)
C2—N1	1.466 (2)	C7—H7A	0.9600
C3—C4	1.370 (3)	C7—H7B	0.9600
C3—H3	0.9300	C7—H7C	0.9600
C4—C5	1.371 (3)	N1—O2	1.2200 (18)
C4—H4	0.9300	N1—O3	1.2217 (18)
			
C2—C1—C6	116.51 (13)	C6—C5—H5	120.0
C2—C1—C1^i^	123.77 (15)	O1—C6—C5	124.06 (15)
C6—C1—C1^i^	119.63 (14)	O1—C6—C1	115.19 (13)
C1—C2—C3	123.51 (15)	C5—C6—C1	120.75 (15)
C1—C2—N1	119.86 (13)	O1—C7—H7A	109.5
C3—C2—N1	116.60 (14)	O1—C7—H7B	109.5
C4—C3—C2	118.11 (16)	H7A—C7—H7B	109.5
C4—C3—H3	120.9	O1—C7—H7C	109.5
C2—C3—H3	120.9	H7A—C7—H7C	109.5
C3—C4—C5	121.01 (15)	H7B—C7—H7C	109.5
C3—C4—H4	119.5	O2—N1—O3	123.39 (16)
C5—C4—H4	119.5	O2—N1—C2	119.06 (13)
C4—C5—C6	120.08 (17)	O3—N1—C2	117.55 (16)
C4—C5—H5	120.0	C6—O1—C7	118.46 (14)
			
C6—C1—C2—C3	0.7 (2)	C2—C1—C6—O1	178.01 (14)
C1^i^—C1—C2—C3	177.26 (13)	C1^i^—C1—C6—O1	1.26 (19)
C6—C1—C2—N1	178.92 (14)	C2—C1—C6—C5	−1.6 (2)
C1^i^—C1—C2—N1	−4.5 (2)	C1^i^—C1—C6—C5	−178.36 (13)
C1—C2—C3—C4	0.6 (3)	C1—C2—N1—O2	−36.5 (2)
N1—C2—C3—C4	−177.73 (16)	C3—C2—N1—O2	141.90 (16)
C2—C3—C4—C5	−0.9 (3)	C1—C2—N1—O3	144.16 (17)
C3—C4—C5—C6	0.0 (3)	C3—C2—N1—O3	−37.5 (2)
C4—C5—C6—O1	−178.25 (16)	C5—C6—O1—C7	−4.5 (3)
C4—C5—C6—C1	1.3 (3)	C1—C6—O1—C7	175.90 (18)

Symmetry codes: (i) −*x*+1, *y*, −*z*+3/2.

### Hydrogen-bond geometry (Å, °)

**Table:**

*D*—H···*A*	*D*—H	H···*A*	*D*···*A*	*D*—H···*A*
C7—H7B···O3^ii^	0.96	2.48	3.426 (3)	169

Symmetry codes: (ii) *x*−1/2, *y*−1/2, *z*.
